# Biosurfactants produced by *Scheffersomyces stipitis* cultured in sugarcane bagasse hydrolysate as new green larvicides for the control of *Aedes aegypti*, a vector of neglected tropical diseases

**DOI:** 10.1371/journal.pone.0187125

**Published:** 2017-11-10

**Authors:** Paulo Ricardo Franco Marcelino, Vinícius Luiz da Silva, Rafael Rodrigues Philippini, Cláudio José Von Zuben, Jonas Contiero, Júlio César dos Santos, Silvio Silvério da Silva

**Affiliations:** 1 Department of Biotechnology, Engineering School of Lorena, São Paulo University, Lorena, Brazil; 2 Department of Biochemistry and Microbiology, Biosciences Institute, São Paulo State University (Campus Rio Claro), Rio Claro, Brazil; 3 Department of Zoology, Biosciences Institute, São Paulo State University (Campus Rio Claro), Rio Claro, Brazil; Natural Resources Canada, CANADA

## Abstract

Biosurfactants are microbial metabolites with possible applications in various industrial sectors that are considered ecofriendly molecules. In recent years, some studies identified these compounds as alternatives for the elimination of vectors of tropical diseases, such as *Aedes aegypti*. The major bottlenecks of biosurfactant industrial production have been the use of conventional raw materials that increase production costs as well as opportunistic or pathogenic bacteria, which restrict the application of these biomolecules. The present study shows the potential of hemicellulosic sugarcane bagasse hydrolysate as a raw material for the production of a crystalline glycolipidic BS by *Scheffersomyces stipitis* NRRL Y-7124, which resulted in an emulsifying index (EI_24_) of 70 ± 3.4% and a superficial tension of 52 ± 2.9 mN.m^-1^. Additionally, a possible new application of these compounds as biolarvicides, mainly against *A*. *aegypti*, was evaluated. At a concentration of 800 mg.L^-1^, the produced biosurfactant caused destruction to the larval exoskeletons 12 h after application and presented an letal concentration (LC_50_) of 660 mg.L^-1^. Thus, a new alternative for biosurfactant production using vegetal biomass as raw material within the concept of biorefineries was proposed, and the potential of the crystalline glycolipidic biosurfactant in larvicidal formulations against neglected tropical disease vectors was demonstrated.

## Introduction

Biosurfactants (BS) are microbial metabolites with amphiphilic structures that exhibit tensioactive, emulsifying, antimicrobial and antitumoral properties and are considered to be biodegradable and non-toxic, making them ecofriendly products compared to the synthetic surfactants widely used by modern societies [1, 2]. These bioproducts are commonly produced by bacteria, such as *Pseudomonas* and *Bacillus*, that are opportunistic species or pathogenic to various organisms. Recently, yeasts have been frequently used as fermenting agents of BS because many of them have GRAS (Generally Recognized as Safe) status, which eliminates the risks associated with toxicity and pathogenicity and allows applications in the food and pharmaceutical industries without restrictions [3, 1]. In addition, the production of BS using industrial by-products in bioprocesses is considered advantageous over synthetic routes given the current required sustainability demands and reductions in production costs. Due to their advantages and physicochemical and biological properties, BS are considered versatile molecules that can be applied in the chemical, petroleum, food, pharmaceutical, cosmetic and agricultural industries [4, 5].

In recent years, agriculture has used BS as emulsifiers in various herbicidal and insecticidal formulations. In addition, studies have reported that some glycolipids (sucrose esters) decrease survivorship and oviposition in the mite *Calacarus heveae* Feres—Acari: Eriophyidae, causing death [6]. Recently, the insecticidal effect of BS has also been explored against vectors of tropical diseases, such as *Aedes* (*Stegomyia*) *aegypti* (Diptera: Culicidae) (Linnaeus, 1762), and this application is of extreme interest for public health programs aimed at controlling such urban pests [7, 8].

*Aedes aegypti* has been disseminated across the planet since the XVII^th^ Century and has been reported on every continent, particularly in countries with tropical and subtropical climates. This Culicidae mosquito is responsible for the transmission of tropical arboviruses, such as dengue, yellow fever, Zika and chikungunya, which have been causing worldwide concern in recent years. In Brazil, in 2016, 1.5 million probable cases of dengue, 263,598 cases of chikungunya and 211,770 cases of Zika were reported. Zika is also associated with 1,750 confirmed cases of microcephaly in newly born children as well as the intensification of some degenerative ailments such as *Guillain-Barré* syndrome [9–11]. Several synthetic insecticides, such as organochlorines, organophosphates, carbamates and pyrethroids, are used for the control of *A*. *aegypti*, but the toxicity of these compounds and the acquired resistance that mosquitoes present over time necessitates the replacement of these insecticides [12].

Due to their sustainable appeal and the advent of microbial biotechnology and green chemistry, the prospecting of natural substances and methods for the elimination of *A*. *aegypti* has been increasing as shown by the adoption of toxins from entomopathogenic bacteria, insect growth regulators (IGRs) and plant extracts [13–15]. In addition, these agents are applied as larvicides, which is one of the most used means of controlling the mosquito population [16].

Based on the environmentally friendly features of BS in addition to their larvicidal and mosquitocidal properties, this study aimed to evaluate the larvicidal potential of BS produced by yeasts using sugarcane bagasse hemicellulosic hydrolysate as the carbon and energy source against *A*. *aegypti*.

This work will contribute to the introduction of these products as green and efficient alternatives for combatting neglected tropical diseases that ravage people in several parts of the world. Sugarcane bagasse was chosen as the raw material due to its availability in certain countries, such as Brazil and India [17]. The use of the hemicellulosic fraction to obtain the BS was emphasized in the present work because the integral exploitation of lignocellulosic biomass is needed to consolidate biorefineries [18].

## Materials and methods

### Raw material and chemicals

Sugarcane bagasse (SB) was obtained from Usina Costa Pinto/COSAN (Piracicaba, SP, Brazil). Commercial 98% sulfuric acid purchased from Fluka/Sigma Aldrich (St. Louis, Missouri, USA) was used for hydrolysis of the SB. The granular activated charcoal used in the detoxification step was obtained from Alfa LU 10X30 (Brasilac, Brazil). Yeast extract, malt extract and bacteriological peptone were purchased from Sigma-Aldrich (St. Louis, Missouri, USA). Commercial kerosene was purchased at a local market. All other chemicals were of analytical grade.

### Obtaining the sugarcane bagasse hemicellulosic hydrolyzate by acid hydrolysis

Hemicellulosic hydrolyzate was obtained using a 30-L stainless steel reactor loaded with SB and sulfuric acid (100 mg acid/g of SB) added in a solid/liquid proportion of 1:10 and operated at 121°C for 15 min [19]. After the hydrolysis step, the hemicellulosic hydrolyzate was concentrated in a 30-L vacuum evaporator at 70°C until the xylose content reached 70 g.L^-1^. The concentrated hydrolyzate was detoxified using an overliming technique combined with addition of 1% activated charcoal (detoxified hydrolyzate—DH) [20]. The sugar (xylose, arabinose and glucose) (g.L^-1^) and furan (furfural and 5-hydroximethyl-furfural) (mg.L^-1^) contents in the DH were quantified using High Performance Liquid Chromatography (HPLC), and the total phenolic compounds (g.L^-1^) were measured by a spectrophotometric method at 725 nm [21]. The DH was stored at -20°C to prevent the possible degradation of sugars as well as contamination.

### Maintenance of the yeast strain and BS production

The strain *Scheffersomyces (Pichia) stipitis* NRRL Y-7124 was obtained from the Collection of the Northern Regional Research Laboratory (Peoria, Illinois, United States of America). Petri dishes with Yeast Malt Agar medium (YMA) (g.L^-1^: glucose, 10; yeast extract, 3; malt extract, 3; and bacteriological peptone, 5) were used to maintain the strain, and after 48 h of static growth in a bacteriological incubator at 30.0 ± 2.0°C, the cultures were transferred to a refrigerator at 4°C.

For inoculum preparation and fermentation a mineral medium (g.L^-1^: potassium dihydrogenphosphate, 2.0; yeast extract, 10.0; ammonium nitrate, 11.0; magnesium sulfate heptahydrate, 2.0; and carbon source, 40.0) supplemented with pure xylose (SX medium) or detoxified hemicellulosic hydrolyzate (DH medium) as a carbon source was used. The pH of the media was adjusted to 5.0–5.5 [22].

For inoculum preparation, one loopful (3 mm) of the yeast strain was transferred to a 50.0-mL Erlenmeyer flask containing 10.0-mL of the SX medium. The flask was incubated at 30°C with stirring at 200 rpm for 48 h in a rotary shaker. After incubation, the cells were isolated by centrifugation (12000 x g, 10 min) and resuspended in 0.9% (w/v) sterile saline solution. Approximately 1 mL of cell suspension was inoculated into the fermentation flasks to obtain an initial absorbance of 1.0 at 600 nm, corresponding to 1.0 x 10^7^ total cells.mL^-1^.

Fermentation assays were performed in triplicate in 1.0-L Erlenmeyer flasks containing 200 mL of DH medium and were incubated at 30°C with stirring at 200 rpm for 68 h in a rotary shaker. After fermentation, the cultures were centrifuged at 12000 x g for 10 min to remove the cells. BS containing supernatant was used for the isolation and characterization procedures.

### BS isolation and partial characterization

For BS precipitation and isolation, cell-free culture broth was acidified with 6 M HCl to pH 2.0 and refrigerated for 12 h at 4°C. Then, precipitated BS was extracted with an equal volume of ethyl acetate (1:1, v/v); this procedure was repeated 3 times. The organic extract was dried at 37°C for 72 h until the residual solvent evaporation, and the BS was resuspended in 0.01 mol.L^-1^ phosphate buffer and kept under refrigeration (4°C) [23].

Spectrophotometric tests for total and reducing sugars at 490 nm [24, 25], total lipids at 530 nm [26] and total protein at 595 nm [27] were performed for crystallized BS characterization.

#### Glycolipids test

To identify the BS produced by *S*. *stipitis* NRRL Y-7124 in the DH medium, a test for glycolipid confirmation was performed that involved acid precipitation in chloroform followed by reaction with methylene blue [28].

#### Thin layer chromatography (TLC)

TLC of the organic extract was performed on silica gel 60 F254 aluminum-coated sheets (Merck, Darmstadt, Germany) using as the eluent a mixture of chloroform: methanol: distilled water (65:15:1 v/v). The spots were observed under visible light after treatment with a “Seebach staining solution” [29].

#### Fourier transform infrared spectroscopy (FTIR)

FTIR analysis of the purified BS was carried out using a spectrometer (DX Perkin Elmer, Waltham, Massachusetts, USA) with potassium bromide (10 mg of sample and 90 mg of potassium bromide) as support in a wavenumber range of 4000–400 cm^-1^.

#### X-ray diffraction of the BS crystals

X-ray diffraction measurements were carried out with an Empyrean diffractometer (PANalytical, Almelo, The Netherlands) equipped with a X’Celerator X’Pert detector. A Cu anode was used as the X-ray source (K radiation: 40 kV and 40 mA), and a 1/4° divergence slit was used to collect the data in the 2θ range of 10–80°.

#### Emulsifying and tensioactive properties

The emulsifying index (EI_24_) was determined by the addition of 2 mL of kerosene to the same volume of cell-free culture broth supernatant in glass tubes. The tubes were mixed by vortexing at 9000 rpm for 1 min and were incubated at room temperature for 24 h. The stability of the emulsion was determined after 24 h, and the EI_24_ was calculated as described previously [30]. Surface tension (ST) measurements were performed using a tensiometer (Kruss FM40 Easy drop, Hamburg, Germany) with the DropShape Analysis software using the Pendant Drop method.

#### Acid hydrolysis of crystalized BS

Acid hydrolysis of crystalized BS was performed by reacting 10 mg of BS with 1 mL of a 5% HCl-methanol solution for 12 h at room temperature. Then, the reaction was quenched by adding 1 mL of water. The methylated lipids present in the mixture were removed by liquid-liquid extraction using hexane. The water-soluble fraction was then neutralized using a 0.001 mol/L NaOH solution to pH 6.0–7.0, followed by HPLC analysis [31].

#### HPLC analysis

The glucose, xylose, arabinose and acetic acid concentrations were determined by high-performance liquid chromatography (HPLC) (Agilent Technologies 1200 Series, Santa Clara, USA) using a Bio-Rad AMINEX HPX-87H (300 x 7.8 mm) column at 45°C with a refraction index detector (RID6A). Sulfuric acid (0.01 N) was used as the eluent with a flow rate of 0.6 mL/min, and the injection volume was 20 μL.

### Larvicidal activity tests

#### Egg acquisition

The eggs of *A*. *aegypti* mosquito specimens were provided by Merieux NutriSciences (Charqueada–SP, Brazil). The eggs were placed in 40 mL of mineral water to hatch. After hatching, the larvae were fed, and when they reached the third instar of development, they were transferred to flasks for the larvicidal experiments [8].

#### Larvicidal evaluation

A total of 40 mL of either BS or the control solution (mineral water) were added to 60-mL flasks with 10 *A*. *aegypti* larvae in the third instar of development. The flasks had small holes in the caps to allow gas exchange. The tests were performed using solutions containing 50, 100, 200, 400, 800 or 1000 mg/L BS in addition to the control. Larvicidal activity was quantified based on the time of permanence at the surface, the number of attempts to stay on the surface and the number of dead larvae. The quantifications were done in real time; the larvae were considered dead when they did not show any signs of activity or movement. The experiments were carried out in triplicate.

### Microscopy analysis

Images of the BS crystals and mosquito larvae were acquired using an optical microscope (DMLB, Heidelberg, Germany) with a digital camera (Leica DC100) at 100 - 400X magnification.

### Statistical analysis

All of the fermentations and quantitative tests were performed in triplicate. The results are expressed as the mean with the arithmetic standard deviation calculated. The Reed-Muench (Behrens) and intersection curve methods were used for the DL_50_ calculations [31, 32]. *GraphPad Prism 5*.*0* was used for the calculations.

## Results and discussion

### Characterization of hemicellulosic sugarcane bagasse hydrolyzate and BS production

The detoxified hemicellulosic sugarcane bagasse hydrolysate (DH) used in this study had the following composition: 58.764 ± 1.076 g.L^-1^ xylose, 4.300 ± 0.053 g.L^-1^ arabinose, 3.677 ± 0.076 g.L^-1^ glucose, 0.047 ± 0.009 mg.L^-1^ total phenolics and 0.123 ± 0.000 mg.L^-1^ furans (furfural and HMF). After the addition of nutrients to the DH, the medium for BS production had the following composition: 40 ± 2.69 g.L^-1^ xylose, 2.7 ± 0.029 g.L^-1^ arabinose and 1.34 ± 0.019 g.L^-1^ of glucose. Consequently, the compounds considered toxic to cellular metabolism, furans and phenolics, were diluted to 0.028 ± 0.006 and 0.91 ± 0.003 mg.L^-1^, respectively. In the literature, there are no reports of furans and phenolic compounds influencing BS production by yeasts, but some papers have explored their effects on the production of other metabolites. A study on ethanol production showed that concentrations of up to 0.30 g.L^-1^ of furans (furfural and HMF) did not inhibit cell growth nor ethanol production by *S*. *stipitis* NRRL Y-7124 [33]. In studies with *Candida guilliermondii* for xylitol production, analyses of the effects of phenolic compounds of low molar mass (vanillin and syringaldehyde) were performed individually and in combination. According to the authors, 2.0 g.L^-1^ vanillin inhibited microbial growth by 23%; however, xylitol productivity (0.62 g.L^-1^.h^-1^) was not affected. A concentration of 2.0 g.L^-1^ syringaldehyde inhibited cell growth by 75%, yielding 0.14 g.L^-1^.h^-1^. Moreover, the combination of vanillin and syringaldehyde, both at concentrations of 1.0 g.L^-1^, showed a 50% reduction in xylitol productivity (0.3 g.L^-1^.h^-1^) [34].

After 68 h of cultivation, *S*. *stipitis* NRRL Y-7124 consumed approximately 60% of the xylose and 100% of the glucose present in the DH medium, but the concentration of arabinose did not vary until the end of the fermentation. The BS quantification tests showed a production of 0.70 ± 0.030 g.L^-1^. Analysis of the furans and phenolic compounds showed that the furans were completely consumed, and approximately 10% of the phenolics remained in the medium. The metabolism of phenolic compounds by *P*. *stipitis* is due to the presence of the cytochrome P450 enzyme complex, which is contains redox enzymes such as oxygenases that oxidize xenobiotic compounds (furans, phenolics, hydrocarbons and others) [35]. These results are similar to a recent study that reported the production of BS by *Candida bombicola* in a medium with acid cellulosic/hemicellulosic corn fiber hydrolysate, obtaining 1.000 g.L^-1^ of sophorolipid after 192 h of fermentation [36]. The authors also observed the consumption of furans and other compounds, with HMF totally metabolized after fermentation.

### BS characterization

In this study, the BS produced by *S*. *stipitis* NRRL Y-7124 after 68 h of cultivation was characterized by spectroscopic methods (UV-Vis and FTIR), TLC and X-ray diffraction and was evaluated for its emulsifying and tensioactive properties as well as its composition of monosaccharides in the glycidic fraction.

The EI_24_ and ST measurements of the free cell supernatant in kerosene were 70 ± 3.4% and 52 ± 2.9 mN.m^-1^, respectively. The results showed that the BS produced in the DH medium had a better emulsifying property than tensioactive characteristic, probably due to the BS molecule size and type [37]. The EI_24_ results obtained are similar to or greater than others studies of BS produced by yeasts with different carbon sources, which reported EI_24_ measurements ranging from 40–76% [38; 39]. The emulsifying property of the BS produced in the present work in hydrophobic compounds shows its potential for future applications in bioremediation processes as well as cosmetic, agricultural, and food formulations [40].

**Fig 1:**
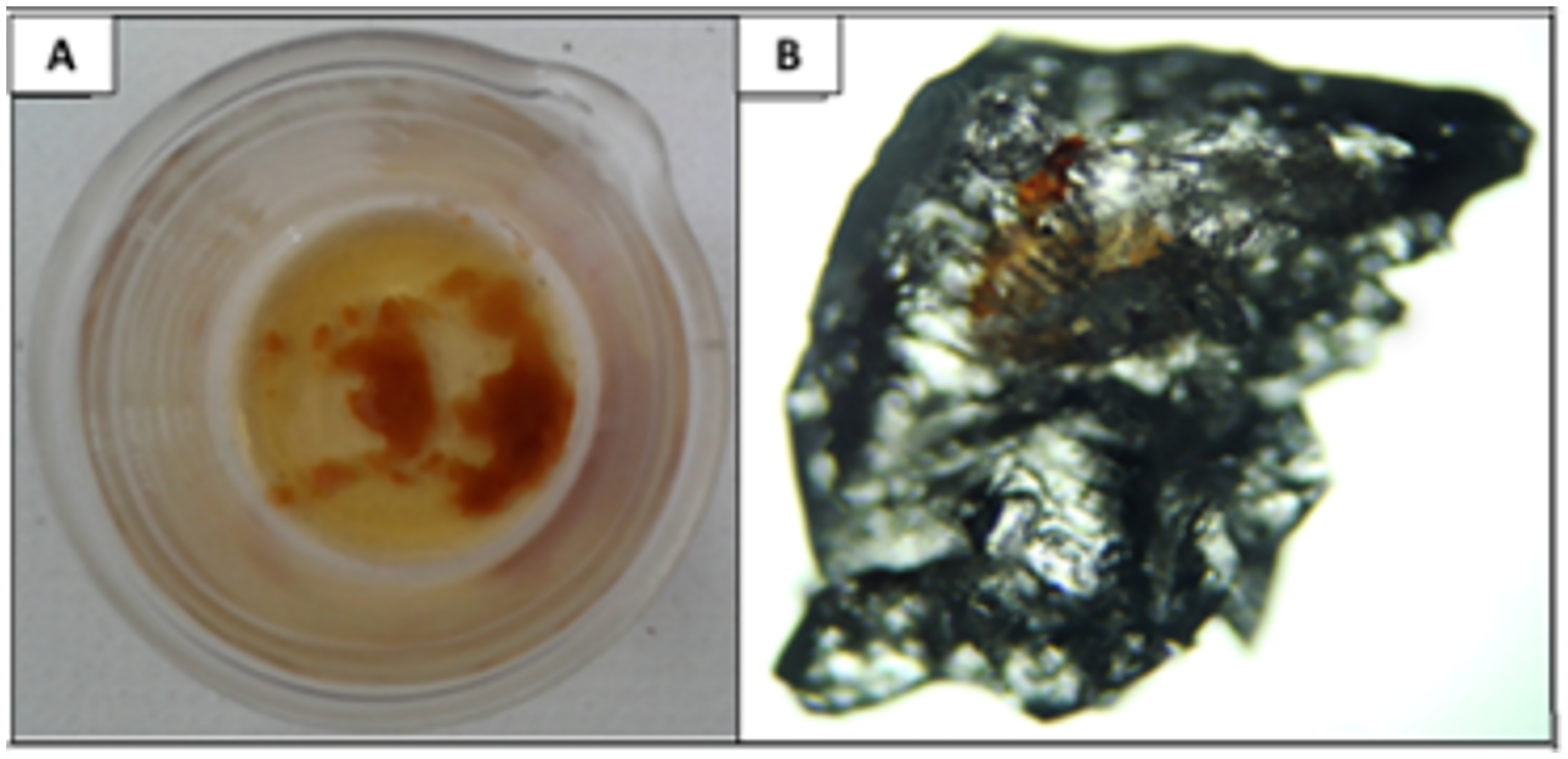
**(A)** Photography of the BS produced by *S*. *stipitis* NRRL Y-7124 in the HD medium after extraction and evaporation of the solvent. **(B)** Optical micrograph of the crystallized BS produced by *S*. *stipitis* NRRL Y-7124 in the HD medium (magnification of 1000X).

After extraction, an oily yellow/brownish crude BS (Fig 1A) was obtained and resuspended in phosphate buffer. After standing for crystallization, the crystals that formed (Fig 1B) were qualitatively characterized for the presence of total and reducing sugars, proteins and lipids. The analyses showed a non-reducing sugar composition of 32% and a lipid content of 64%, indicating the probable production of a BS glycolipid by *S*. *stipitis* NRRL Y-7124.

The reaction of BS with blue methylene in an organic medium resulted in an absorbance of 0.425 (738 nm), evidencing the presence of glycolipidic BS. The blue color obtained in the reaction is due to the complexation between the carboxyl groups of the glycolipids and the methylene blue dye. This test has been used in experiments on rhamnolipids produced by *Pseudomonas* and *Bacillus* [28].

**Fig 2:**
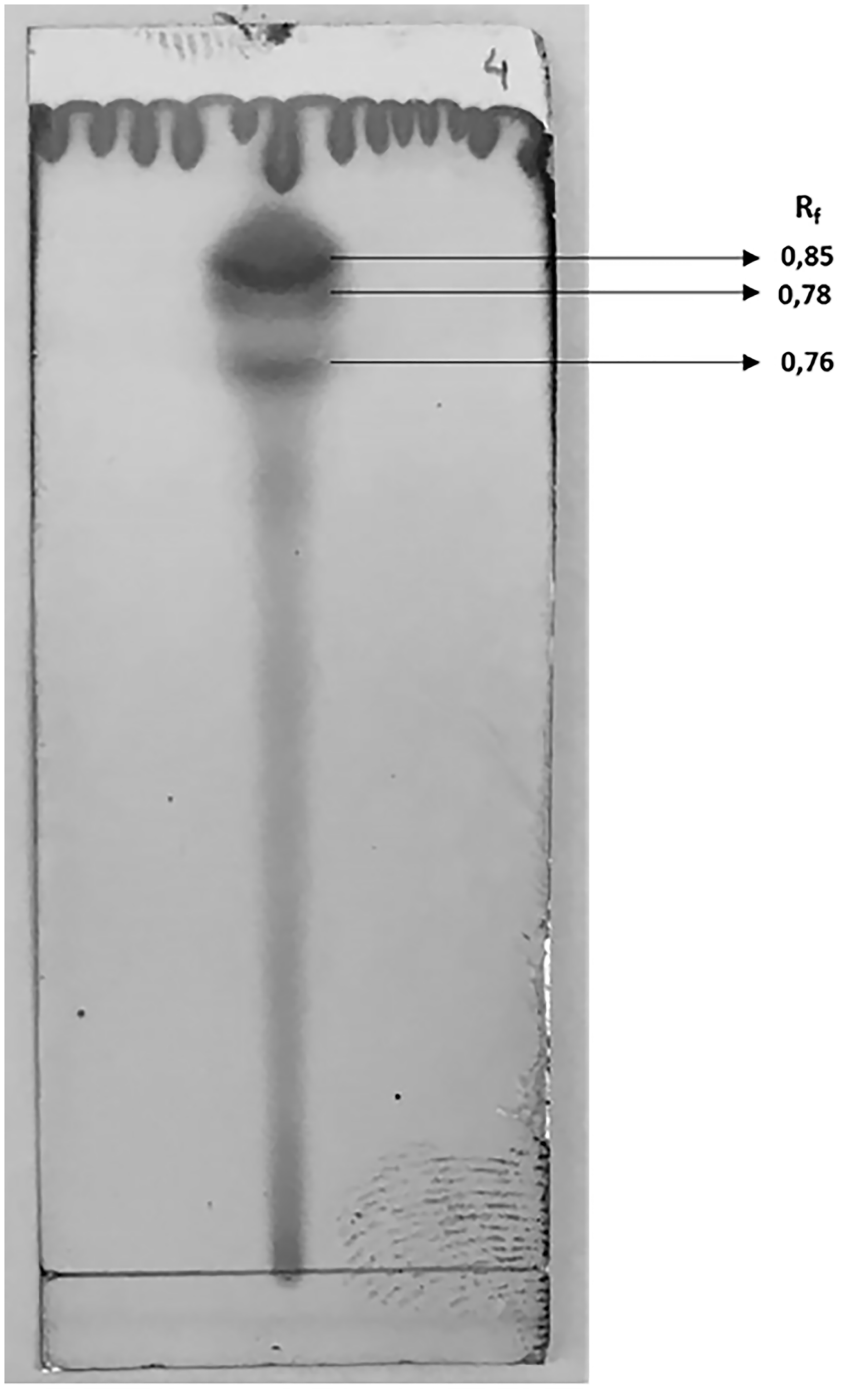
TLC analysis of the BS produced by *S*. *stipitis* in the DH medium.

TLC analysis of the BS produced in the HD medium showed spots with retention factors (R_f_) of 0.76, 0.78 and 0.85, indicative of glycolipid production (Fig 2). In studies of BS secreted by *Cyberlindnera samutprakarnensis* JP52 various sophorolipids with different R_f_ values, which ranged from 0.6 to 0.9 due to size of the carbonic chain in their apolar regions, were reported [41].

**Fig 3:**
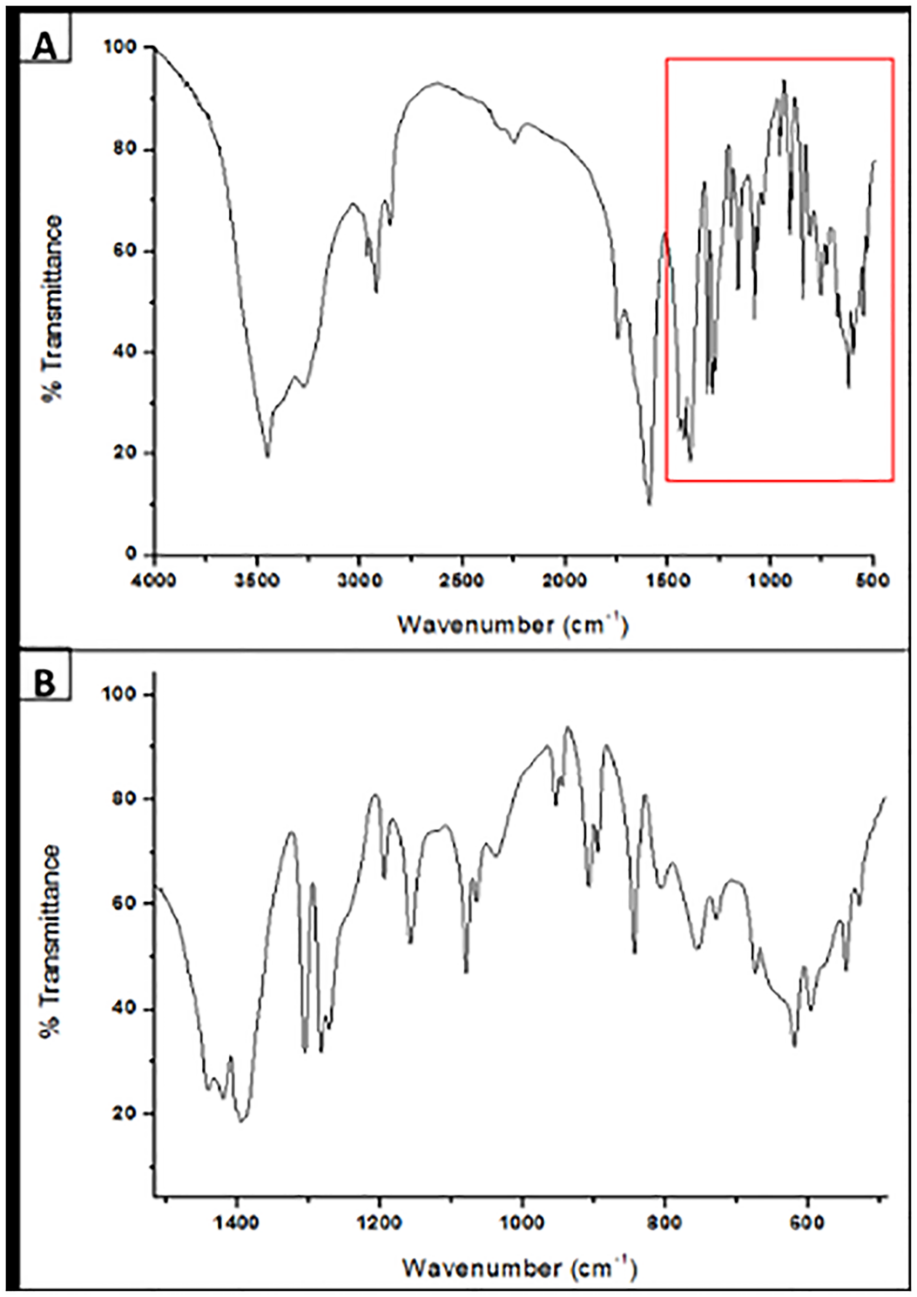
FTIR of the crystallized BS produced by *S*. *stipitis* NRRL Y-7124 in the DH medium. **(A)** FTIR spectrum with wavenumbers between 402–4000 cm^-1^, and **(B)** FTIR spectrum with wavenumbers in the fingerprint region between 402–1716 cm^-1^.

FTIR analysis (Fig 3A and 3B) shows stretching bands for hydroxyl (R-OH) groups between 3200–3400 cm^-1^, with the typical pattern for carboxylic acids and axial deformation peaks for C-H bonds. Asymmetrical stretching of methylene (*V*_*as*_ CH_2_) and methyne (*V*_*as*_ CH) occurred at 2919–2953 cm^-1^. Near 1600 and 1200 cm^-1^, the presence of stretching peaks for carbonyl groups (R-C = O) and absorption bands from acetyl esters were observed (Fig 3A). The peaks in the region near 1450 cm^-1^ probably correspond to the C-O-H in the plane binding of carboxylic acid (R-COOH) (Fig 3B). The bands present between 617 and 1460 cm^-1^ (fingerprint region) represented vibrational deformations of R-C-H, R-C-O-H and R-O-H linkages typical of carbohydrates (Fig 3B) [42]. The bands and peaks reported in this study are commonly found in glycolipids produced by yeasts and bacteria [43, 44, 45].

**Fig 4:**
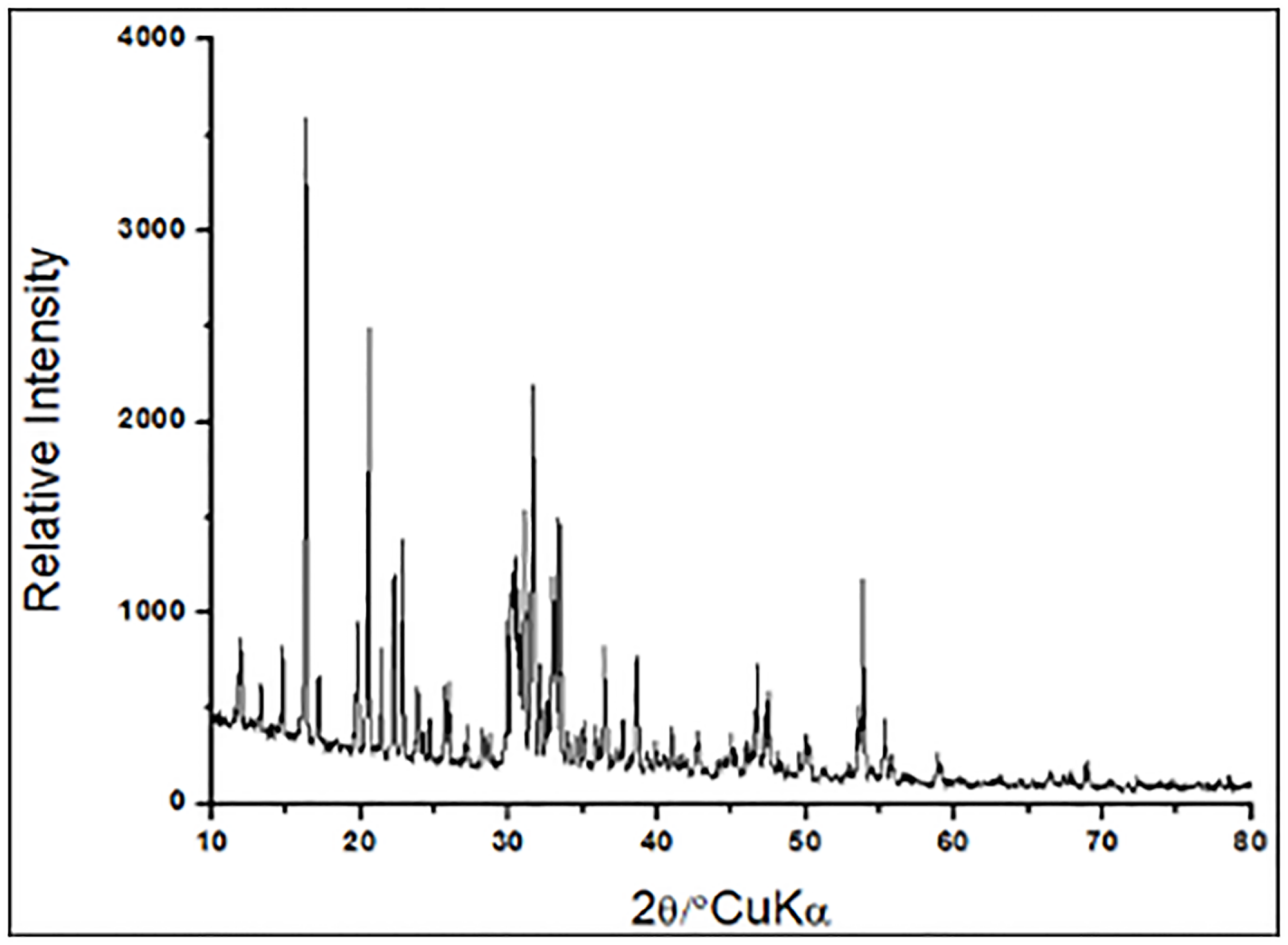
X-ray diffractogram of the crystallized BS produced by *S*. *stipitis* NRRL Y-7124 in the DH medium.

X-ray diffraction of the BS crystals (Fig 4) showed an organized crystalline structure, as evidenced by the intense peaks in the 10–55° region. Similar results were found for poly-sophorolipids synthesized chemically, whose diffraction showed intense peaks in the region between 3.6–33° [46]. This crystal structure was also observed for rhamnolipidic BS with a rhomboidal aspect that was produced by *Pseudomonas* [47].

**Fig 5:**
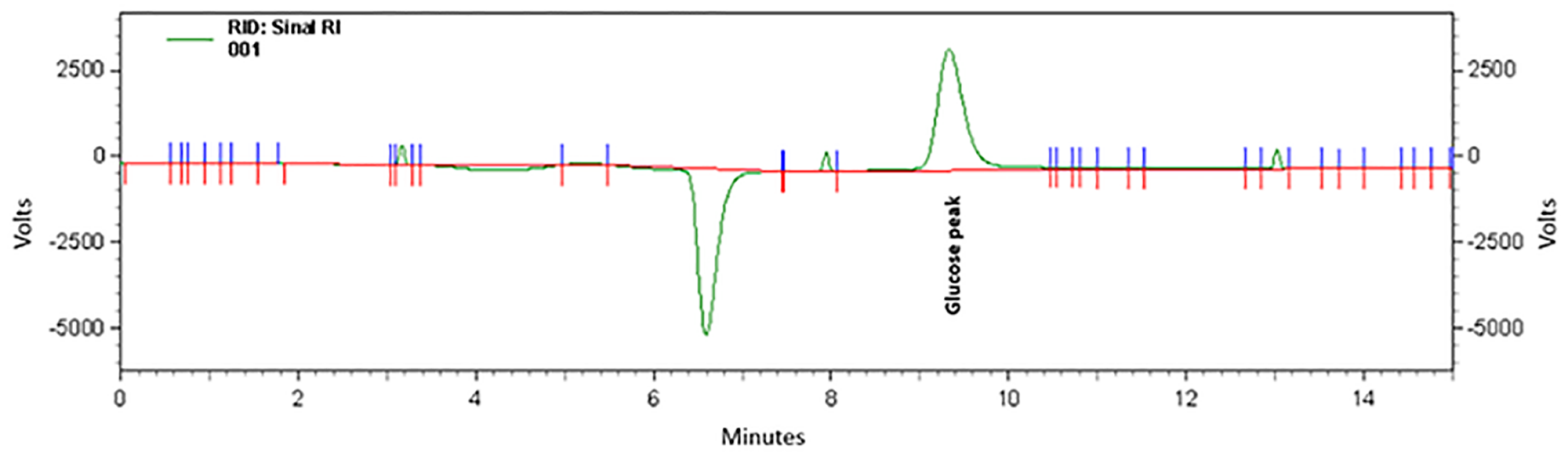
HPLC chromatogram of the monosaccharides in the crystallized BS produced by *S*. *stipitis* in the DH medium.

The crystallized BS was hydrolyzed, and the products present in the ethyl acetate extract were analyzed by HPLC. The presence of a single peak with a retention time (t_r_) of 9.50 min corresponding to glucose indicated that the glycidic fraction of the glycolipid could be cellobiose or sophorose (Fig 5). Actually, cellobiose lipids or sophorolipids are glycolipidic BS commonly secreted by yeasts [47–49].

### Application of BS as larvicide against *A*. *aegypti*

The BS glycolipid produced by the yeast was tested as a larvicide against *A*. *aegypti*, a vector of many neglected tropical diseases that have caused various problems to humans in recent years [11].

The produced and purified BS was tested at different concentrations against *A*. *aegypti* larvae standardized in the third stage of development, a larval period in which there is intense metabolic activity and greater larvicidal resistance. As shown in Table 1, 12 h after the application of the BS at concentrations of 800 and 1000 mg.L^-1^, 100% of the larvae died. In the tests with a BS concentration of 400 mg.L^-1^, only 20% of the larvae died after 24 h, whereas for concentrations less than 400 mg.L^-1^, death was not observed; however, motility decreased, and the larvae remained submerged for longer periods of time. Subsequently, the LC_50_ of the BS was calculated to be 660 mg.L^-1^.

**Table 1:** Number of live *A*. *aegypti* larvae 96 h after the application of different glycolipidic BS concentrations produced by *S*. *stipitis* NRRL Y-7124 in the HD medium: control, 400, 800, and 100 mg.L^-1^.

Glycolipidic BS concentration (mg.L^-1^)	Initial number of larvae	Average number of live larvae	Standard deviation	% Elimination
**Control**	20	20	0	0
**400**	20	15	0,707	80
**800**	20	0	0	100
**1000**	20	0	0	100

Beginning 3 h after the application of BS at concentrations of 800 and 1000 mg.L^-1^, the larvae exhibited an unusual behavior of intense agitation and did not maintain their usual positions near the water surface, which is where they carry out the gas exchange. Studies with *A*. *aegypti* larvae have reported that the respiratory siphon has an internal region that is naturally highly hydrophobic, guaranteeing humidity control due to its chemical composition, which is rich in waxy lipids. BS application favors the interaction of the apolar region of the molecule with the respiratory siphon, which exposes the polar portion of the BS, and consequently, the siphon internal surface becomes polar. Thus, water flows through the spiracular cavity of the larvae, killing them by drowning. In addition, tracheal air sacs, which present a specific gravity very close to that of water and facilitate ascent to the surface for respiration, are influenced by the presence of BS, altering and disturbing the hydrostatic equilibrium of the larvae, leading to an exacerbated energy expenditure and exhaustion, which assist in death by drowning [50].

In the literature, there are reports of microbial BS that have been tested as larvicidal and pupicidal agents. The efficacy of these compounds as agents for the control of vectors of neglected tropical diseases, usually expressed as LC_50_ values, varies according to the type (chemical structure) of BS, the time of application (contact between larvae or pupae) and some physical properties (temperature and pH of the medium). In some reports, the LC_50_ values were lower than that observed in our work. Some studies reported that cyclic lipopeptides produced by *Bacillus subtilis* showed LC_50_ values ranging from 5.57 to 10.6 mg.L^-1^ against *Anopheles stephensi* larvae, and surfactin produced by *Bacillus sphaericus* exhibited LC_50_ values of 2.2 mg.L^-1^ at neutral pH and 3.0 mg.L^-1^ at pH 11 (Manonmani et al., 2011; Geetha et al., 2010). In a more recent study, a rhamnolipid produced by *Stenotrophomonas maltophilia* showed an LC_50_ of 100 mg.L^-1^ after 2 h of application when tested against *Culex quinquefasciatus* larvae, and this value decreased to 30 mg.L^-1^ when the application time was increased to 24 h (Deepali et al., 2014). Conversely, in other recent work, the observed LC_50_ values were similar to or higher than those obtained in our study. In that study of a rhamnolipid produced by *Pseudomonas aeruginosa*, solutions containing 800–1000 mg.L^-1^ of BS killed all of the *A*. *aegypti* larvae after 18 h of application, while solutions containing 400 and 600 mg.L^-1^ exhibited the same effect only after 48 h of application [8].

**Fig 6:**
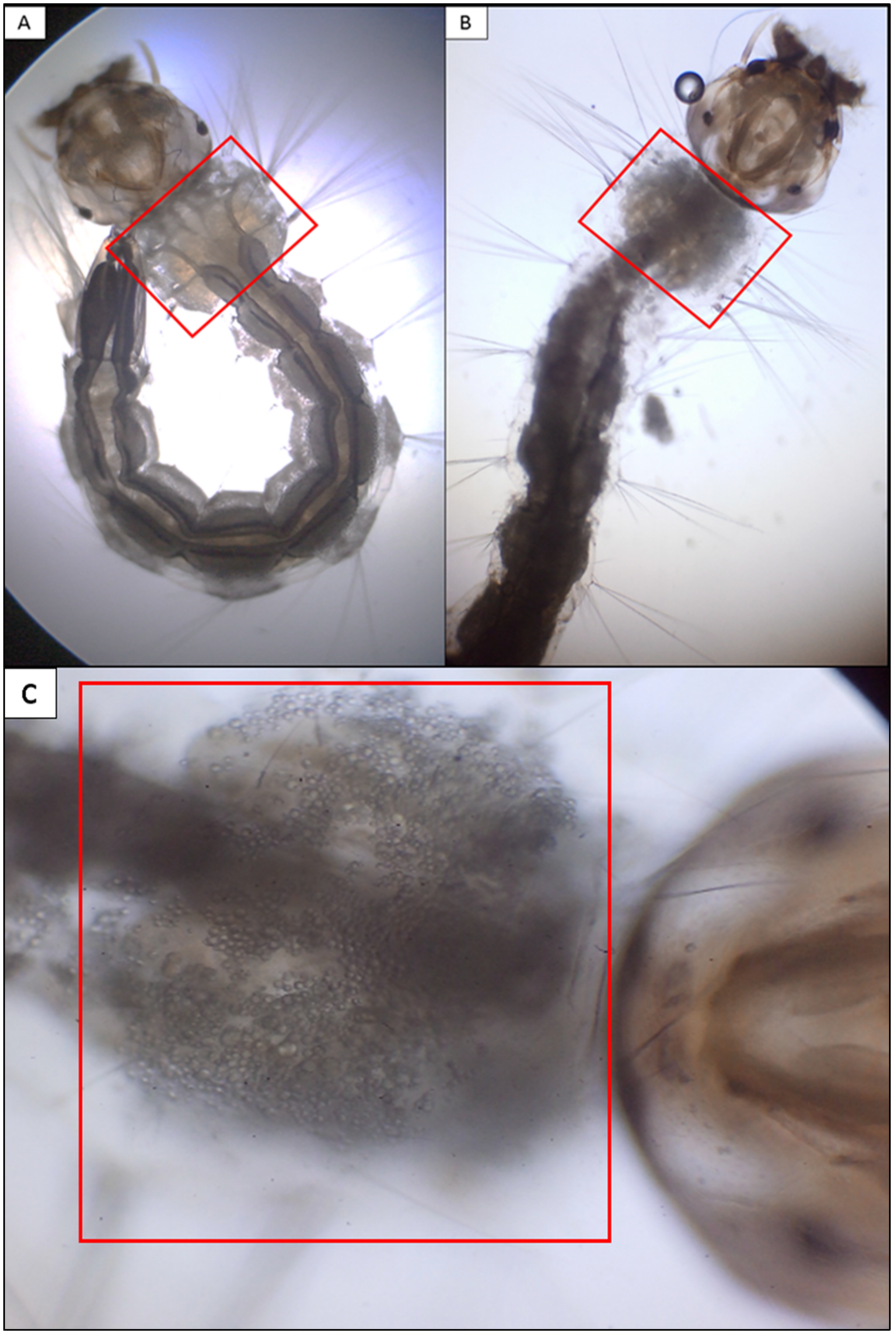
Optical micrographs of *A*. *aegypti* larvae (A) without BS application (control; 400X magnification), (B) 12 h after the application of 800 mg.L^-1^ of the BS produced by *S*. *stipitis* NRRL Y-7124 (400X magnification) and (C) 12 h after the application of 800 mg.L^-1^ of the BS produced by *S*. *stipitis* NRRL Y-7124 (1000X magnification).

Another interesting fact that should be highlighted is the probable effect of the BS on the larva cuticle (Fig 6). After 12 h of BS application (800 mg.L^-1^), decomposition of the larvae bodies was observed, likely due to the emulsification and removal of hydrocarbons and other apolar compounds found on the cuticle (octadecane, n-heneicosane, docosane, nonacosane, long chain carboxylic acids, aldehydes and esters) by the BS [51] (Fig 6B and 6C). Others authors have reported that BS may affect the cells and cuticles of insects that are considered to be agricultural pests or vectors of neglected tropical diseases, causing their death [52, 53, 8]. The continued larval decomposition that occurred with the application of the BS resulted in an inability to observe the larval bodies after 24 h, likely due to the solubilization of chitin, a structural polysaccharide present in the larval exoskeleton.

## Conclusion

The present study reports the use of a BS produced by *S*. *stipitis* NRRL Y-7124 in a medium based on hemicellulosic sugarcane bagasse hydrolysates as a biolarvicide against *A*. *aegypti*, a vector of neglected tropical diseases. Characterization tests showed that the BS produced is a glycolipid, and biological tests revealed the larvicidal potential of this molecule. Another fundamental observation was the quick and efficient mechanism by which the BS eliminated the larvae–through the decomposition of the exoskeleton–which is an unprecedented finding at present in the literature. Based on the results obtained, the present work suggests that BS is a sustainable product for future lignocellulosic biorefineries. In addition, a new application of BS was shown that can help address a public health problem of extreme urgency, reducing and preventing tropical diseases such as *Zika*, which are caused by *A*. *aegypti* and result in fetal deformation and neurological problems such as microcephaly. This work will contribute to the production of this microbial metabolite at a large scale as it illustrates a way to convert alcohol industry residues into a bioproduct of great aggregated value and shows an interesting application of this safe BS.
